# Hospital and Institutional News

**Published:** 1912-06-15

**Authors:** 


### HOSPITAL AND INSTITUTIONAL NEWS.

### THE KING AND QUEEN AT ST. PAUL'S.

As, we trust, no one was allowed to forget, last Sunday was Hospital Sunday, when the King and Queen set an example to all their subjects by attending the morning service at St. Paul's. There was no ceremony, as was the case when in 1903 King Edward and Queen Alexandra were present at the afternoon service at St. Paul's on Hospital Sunday. On this occasion there was no special music, and, with the exception of the choir and of a few reserved seats under the dome, the whole building was, as usual, open to the public. The subject of the Dean's sermon was the parable of the Good Samaritan. The Cathedral was very full, and we can hope only that the rain which deterred nobody from attending at the Cathedral may have proved equally incapable of diminishing the attendance at its daughter churches. The offerings of the congregation at St. Paul's for the Metropolitan Hospital Sunday Fund were taken at the doors.

### AN INFIRMARY SCHEME INVOLVING TOWN PLANNING.

A deputation from the weekly board of the Leeds General Infirmary waited upon the City Council last week in endeavour to enlist the support of the Corporation for the extension scheme which has often been described in The Hospital, and which involves, if completed, a public improvement in the form of a new road sixty feet broad from Calverley Street to Fenton Street. Mr. Charles Lupton, chairman and hon. treasurer of the infirmary, in leading off for the deputation, remarked that the scheme, if adopted, would benefit not only the infirmary but the whole city of Leeds. Indeed, he added, if the infirmary's proposed scheme were not adopted an alternative on similar lines for the city's advantage would soon have to be taken in hand. The area through which the proposed street will pass is practically a slum area. After outlining the extensions, for a summary of which we refer our readers to The Hospital of April 29, 1911 (page 116), Mr. Lupton said that his board was so much convinced of the value of the scheme that the bold step had been taken of purchasing nearly all the land required for it. Sixty properties out of the sixty-six required have now been bought. In short, Mr. Lupton asked were the Corporation prepared to spend £50,000 on land and the construction of the new street and £31,000 on property to the east of it to be used as surplus land? Finally, if the scheme were to be made a true organic whole, the land between the Town Hall and what will be the front of the new infirmary could be cleared for another £50,000, and thus create a splendid open space north of the Town Hall. This, however, is not an integral part of the scheme. A special committee "for dealing with municipal accommodation" is to consider the scheme. The committee consists of Aldermen Lupton, Kinder, Wilson, Hepton, Tetley, Ford, Pickersgill, Midgley, Badlay, and Brown, and Councillors Hodgson, Winn, Lawson, Bowman, Fountain, and Warren. The Deputy Lord Mayor, Alderman W. Penrose-Green, assured the deputation that the subject would receive the fullest consideration of the Council.

### THE LOCAL GOVERNMENT BOARD INQUIRY AT PLYMOUTH WORKHOUSE INFIRMARY.

Mrs. Simpson, Mr. A. W. Lethbridge, and Mr. R. E. Govier, members of the Plymouth Board of Guardians, recently achieved a cherished object, namely, an official inquiry into the administration of Plymouth Workhouse Infirmary. The complaints which have been continually made by certain Guardians and certain patients with reference, in particular, to the care or want of care of those in the consumptive wards, need not be repeated unless the result of the inquiry fails to dispose of them. Suffice it to say that Dr. Fuller, representing the medical department of the Local Government Board, and Mr. E. G. Court, one of the Board's inspectors, visited the infirmary ten days ago and spent the better part of two days in their investigations. The inquiry was private, and pending its published result we make no comment. At the time of writing the vacancy in the post of resident medical officer to the infirmary has not been filled.

### ST. BARTHOLOMEW'S HOSPITAL AND ITS NURSES.

The St. Bartholomew's Hospital dinner at the Mansion House on June 5 will be memorable from the fact that it was given by Sir Thomas Crosby, M.D., F.R.C.S., the first member of the medical profession who has ever held the position of Lord Mayor of London. Sir Thomas Crosby was truly wonderful, for he has proved to be one of the most active, energetic, and able Lord Mayors London has ever had, although he is now upwards of eighty years of age. He has lived a most industrious life, working with the City of London as his centre, having commenced his medical career as house surgeon and subsequently demonstrator of anatomy at St. Thomas's Hospital. Not the least remarkable evidence of his vitality was the energy he put into the last speech made at the dinner, in which he declared, with some emotion, that that dinner would be memorable to him as long as he was spared as one of the most pleasant recollections of the Mansion House, and of his association with the greatest and richest city the world has known. We give a full report of the speeches in another column. It is but just to Lord Sandhurst to congratulate him upon the ability of his speech, which was one of the best delivered and most forcible utterances on the subject of hospitals ever listened to in the Egyptian Hall of the Mansion House. He made a great point of the nurses' accommodation at St. Bartholomew's Hospital, of which we have heard and written much for at least twelve years past. We are convinced that the time has come when the Governors of St. Bartholomew's Hospital should forthwith settle every detail for the new nurses' home, obtain contracts for it, and commence to build without a moment's delay. It is unthinkable that our oldest hospital should continue to permit its nursing staff to be housed so badly, as to make the present accommodation a bye-word throughout the whole hospital world. Every other hospital of importance has long ago made it its business to house its nurses properly. Why should the nurses of St. Bartholomew's Hospital wait? We know no reason, and we are quite sure Lord Sandhurst has the will to put this matter right without a moment's avoidable delay.

### AFTER DINNER ORATORY.

One feature of the St. Bartholomew's dinner at the Mansion House was the speeches of the members of the medical staff. Many new departments have been opened in connection with hospitals and schools during the last decade or two, and we are inclined to think it would be of great service to the medical profession, if another new department were to be added, where the students and even the seniors might accustom themselves to public speaking. They would so acquire the habit of dropping the bedside manner and the colloquial utterance. They might then gain also a voice and manner which would enable any speech which they might have to make distinctly heard when uttered in a public hall or place of assembly. Dr. Samuel West, in order to show that St. Bartholomew's Hospital is not extravagantly managed, gave details of the washing, diets, bandages, and the days of residence of the patients. With reference to bandages, he stated that these formerly were six yards in length, and that the dispenser by reducing the length one yard had prevented much waste and saved the hospital £180 a year on this item alone. Mr. Bruce Clarke, in testimony of the homely and all-embracing character of the work done for the population, gave an account of a patient, a very old man, whose broken bones were mended, whose health was re-established, and who was supplied with a perfect and complete set of new teeth. Thus equipped he was fetched away of the eve of his golden wedding by his wife, who assured the surgeon that he looked handsomer, younger, and better than when she married him fifty years ago. Dr. Norman Moore, the historian of medicine, made a most interesting speech bringing out clearly and forcibly the extraordinary ties which unite most of the great City Companies to St. Bartholomew's Hospital, which have drawn closer and closer the connection between them in the making of the City of London as it is to-day. The clear, incisive, and knowledgeable force of his speech made it a model for similar utterances, and should win for it a place in the records, not only of the hospital but of the City Fathers and the City Companies.

### A DAY OF REST FOR DOCTORS.

We recently had the pleasure of reporting that a plea for one day's rest in seven had been granted to the attendants of a certain mental hospital in Wales. On its heels comes a report from Berlin that the Medical Association is attempting to divide the German capital into districts, each of which will have its own "resident medical officer" on Sunday. The scheme is for the practitioners of each district to take it in turn to act. Whether the report means anything or not it would be interesting to hear what the English medical man would think of a similar proposal to secure him a weekly day of rest.

### THE MERITS OF HEDONAL DISCUSSED IN COURT.

For the last six months or so a drug known as hedonal has been advocated and used for procuring anæsthesia by injection into a vein. For this purpose a 0.75 per cent. solution in normal saline is used. The advantages of being able to procure deep anæsthesia by a method which involves no manipulations about the face at all are obviously great when that region, or the mouth or throat, is the site of operation. Thus for malignant and other growths about the tongue, tonsils, larynx, maxilla, etc., the surgeon is provided with a field of work much less obstructed by anæsthetic apparatus than when the usual inhalation methods are used. That these advantages weigh heavily in favour of hedonal for such special operations cannot be doubted; but it must also be recognised that the risks of the new plan are by no means non-existent, and that at present it is premature to attempt to estimate them. For this reason the "discussion of the merits of hedonal" said to have taken place in Mr. Ingleby Oddie's court at an inquest on a patient who died while being given hedonal is unprofitable; though it is true that the matter thus obtained a publicity in medical circles which possibly it might not otherwise have had. Still the medical press is the best medium for such discussion; and we are glad to note that Messrs. Upcott and Evans have published full details of a similar fatality which occurred at Hull on May 14.

### THE BOTCHING OF A COUNTY HOSPITAL.

As the result of eighteen months' consideration it has been decided by the Governors of the Hertford County Hospital that "a scheme on the lines of Scheme No. 4 be adopted, at a cost not exceeding £10,000, and that a strong committee be formed to raise the necessary funds." In other words, the hospital is not to be scrapped, but to be improved by the erection of new wards and the conversion of the old block into administrative quarters. Scheme No. 4, it may further be explained, is the gist of a preceding scheme costing £12,650, which was itself the outcome of deliberations between Mr. Rowland Plumbe, F.R.I.B.A., and Mr. F. Taylor, A.R.I.B.A., the former of whom was called in in consultation on the plans which Mr. Taylor had drawn up, and added somewhat to them. The recent special meeting revealed two camps among the Governors. Those, like Mr. G. S. Pawle, Mr. J. H. Buxton, and Dr. Shelly, who urged the erection of a new institution; and those others, with whom Mr. Abel H. Smith, the chairman of the Board, appears to sympathise, and including the Rev. O. L. Whitmee, who, though afraid that the £20,000 required for new buildings could not be raised, yet believed that half or one-third would be subscribed for further reconstruction. Mr. Whitmee recalled his committeeman experiences in Sussex, where the money came in easily for a certain reconstruction scheme. It was admitted that the present site is contaminated; it was argued by the medical staff that, as Dr. R. Odell pointed out, no partial scheme touches " one of the most defective parts of the hospital "—the out-patient department; it should be clear, therefore, we think, that a bold effort should be made to provide a new hospital.

### AN OBJECT-LESSON IN HOSPITAL FINANCE.

What Leeds, Chester, and Bradford decided to do three years ago, and are proving capable of doing, Hertford should be able to do also. These Northern cities provide an object-lesson of what the voluntary system can do in the way of finance. The same difficulties faced them, and the same fears were expressed, as at Hertford the other day; and yet what have the last eighteen months proved, and what do the four preceding and severally abandoned schemes really indicate, but the practical impossibility of tinkering with an already unworkable building? Four attempts to patch it have broken down; can those who have the best interests of Hertford Hospital at heart wish a different result to their successor?

### THE SEX QUESTION IN INFIRMARY KITCHENS.

The Bermondsey Board of Guardians have decided to replace a male kitchen staff at their infirmary with a female one. The alteration is expected to save £20 4s. a year, for the present male staff's salaries amount to £369, while the cost of the female staff is estimated at £348 16s. Two reasons are advanced for the change, the first of which is that the cooking is unsatisfactory. For some years this allegation has caused trouble to the Board, and the recent death of the chief male cook gave an opportunity to alter the kitchen staff. The second reason is that the matron can deal more effectively with women than with men. A somewhat serious report from an assistant inspector of the Local Government Board drawing attention to the large number of men sleeping in the institution influenced the Board in coming to a decision. Of the twenty-one single male subordinate officers residing on the premises, ten are now to live out, with a grant of 5s. a week in lieu of lodging. Work will be found in other branches of the service, it is hoped, for the officers displaced by the change.

### A VETERAN SECRETARY.

Since his retirement, already alluded to in The Hospital, the career of Mr. R. A. Owthwaite, the veteran secretary of the City of London Lying-in Hospital, has been the subject of eulogistic comment in the lay press. One City journal, for example, recalls the fact that soon after his appointment as secretary in 1871 it was owing to his influence that an out-patients' department was established. From 1872 to 1907 he was assistant secretary and accountant to the Hospital Sunday Fund, being, therefore, one of the original members of the staff. In view of the King and Queen's visit to St. Paul's last Sunday, it is interesting to recall that Mr. Owthwaite used regularly to be in charge of the Sunday Fund's collections in the cathedral. In 1884 Dr. Heath Strange and Mr. Owthwaite in collaboration established that Home at Hampstead which twenty years later developed into the Hampstead General Hospital. From further facts we note with interest that Mr. Owthwaite's hospital career has been significant of one of the most noteworthy features of hospital work—namely, the number of outside activities in which its best men manage to engage. Thus the secretary to the largest lying-in hospital in London not only found time to associate himself with the allied interests of the then new-founded Sunday Fund and another growing hospital at Hampstead, but in addition acted as private secretary to Sir Polydore de Keyser even during his shrievalty, and, moreover, found official occupation for his Sundays by acting as churchwarden at All Hallows' Church, Hampstead. In addition to this, an enforced holiday in the seventies led Mr. Owthwaite to explore the Transvaal and Natal. Here again, then, work for the voluntary hospitals has proved the starting-point of personal service in very varied fields, and that is perhaps the most fruitful thing to the individual in institutional work.

### SUNDERLAND'S NEW CHILDREN'S HOSPITAL.

The new Children's Hospital, which has been built by the committee of the Royal Infirmary, Sunderland, has been auspiciously opened by Lord Durham. A site of two acres was presented by Mr. John S. G. Pemberton in memory of the late Mr. Richard Pemberton, a former president of the infirmary, and the committee purchased a further two acres of land. Messrs. W. and T. R. Milburn are the architects, and their design includes a central administrative block with two pavilions, having accommodation for fifty-four patients. The pavilions have been named after the late Mr. Frederick Gordon and the late Mrs. John Priestman respectively. Approximately £21,000 has been spent on the site, building, and its equipment, and Lord Durham opened it debt free. Alderman Richardson, chairman of the general committee, and Mr. J. J. Binns requested his lordship to open the building, and Mr. T. Robinson, the secretary, read a statement which Mr. Binns had compiled, setting out the circumstances under which the scheme for a Children's Hospital had been undertaken. The maintenance will still have to be provided, and a league of young people has been founded to give the movement a start. Lord Durham commended the workmen for their representations which had led to the opening of the hospital being hurried on, and closed his speech with a very warm eulogy of Mr. Priestman, to whom, said Lord Durham, the freedom from debt on the opening day was chiefly due. Alderman Richardson further stated that he believed the Royal Infirmary would gain much in popularity from the Children's Hospital, and so long as the workmen continued to provide their generous annual gift of £7,900 he thought the voluntary hospital, and especially the Royal Infirmary, would continue to endure.

### ANOTHER CORONER'S APPOINTMENT.

Dr. A. Douglas Cowburn, M.D., barrister-at-law, has been appointed deputy coroner for the Western District of the County of London. Dr. Cowburn, who took his diploma in 1897 and afterwards gained the M.D. of Brussels, is already medical officer to the Middle and Inner Temples. He studied at St. Thomas's Hospital and at University College, London, and holds the diploma of Public Health. He has published "The Law Relating to the Legal Responsibility of the Medical Man." We are glad to add his name to the list of those recent appointments to the office of coroner which have been given to medical men, though, in the lay press we note that Dr. Cowburn's medical training has not been made as prominent as his legal training. Still the day is now happily past when medical training in candidates for the coronership can be ignored.

### A HOSPITAL OFFICER'S NEW INVENTION.

The pharmacist at the Evelina Hospital for Children, Mr. G. E. Town, M.P.S., is the inventor of an improved vacuum funnel with a side exhaust tube. The idea is the result of investigation and experiments conducted in the laboratory at Guy's Hospital, by the courtesy of Mr. H. Finnemore, chief pharmacist at that institution. We understand that the manufacture will be undertaken by Messrs. Townson and Mercer.

### AN AUSTRALIAN CHECK ON SECRET REMEDIES

Some interesting evidence was given at the last meeting of the Select Committee on Proprietary Remedies by an Australian witness, Mr. H. E. Neale, who was for some years connected with the Customs Office at Melbourne and Sydney. In Australia the proprietary medicine evil is kept in check to some extent by the Commerce Trade Descriptions Act of 1905, which gives the Customs authorities power to prohibit the importation into Australia of goods which are misdescribed. Regulations made under the Act, while they do not require the formulæ of proprietary remedies to be disclosed, provide that if the preparations contain any of the drugs included in a list which forms part of the regulations, the name of the drug and the quantity in the preparation must be mentioned on the label. The witness stated that under this Act in a great many cases—possibly some hundreds—medicines from England had not been permitted entry at Australasian ports until their labels had been amended. Among the descriptions which are false within the meaning of the Act are such expressions as "Nothing in the world so good for you as ——"; "Surpasses anything discovered"; "It cures any disease lurking about the system"; "For all kinds of fevers"; and many other pretentious claims which secret remedy owners in this country are using every day. The weak point is that the Act has no control over goods once they are through the Customs, but fortunately the various Australasian Colonies are favourable to the enactment of legislation embodying the principles of the Commerce Act. Mr. Guy Stephenson, Assistant Director of Public Prosecutions, gave evidence with regard to prosecutions for fraud, and explained the difficulty which is experienced in obtaining the evidence of a complainant in cases where drugs purchased for the purpose of procuring abortion prove to be worthless.

### THE PHARMACEUTICAL SOCIETY'S NEW VICE-PRESIDENT.

Mr. Edmund White, to whom we referred recently as a member of the Pharmaceutical Standing Committee on Insurance, has been elected Vice-President of the Pharmaceutical Society. This honour is well deserved, for Mr. White has had a distinguished career. He was the senior Bell scholar in 1886, took three silver medals at the School of Pharmacy in 1887, and the silver medal of the Pharmaceutical Society in 1888, and became a Fellow of the Institute of Chemistry in 1891. He also graduated at the London University B.Sc. with first-class honours. He was appointed pharmacist at St. Thomas's Hospital in 1889, which post he held until he resigned in order to become general manager to a firm of chemical manufacturers. He is a joint editor of Humphrey and White's "Pharmacopedia" and chairman of the committee which compiles the "British Pharmaceutical Codex."

### AN ALLEGED CUSTOM IN "SLUM PRACTICE."

At one of the recent sessions of the Central Midwives Board a registered midwife was cited to answer a charge of failing, after repeated warnings, to take the temperature of her patients. To this accusation her defence was that she guessed the temperature, "the same as the doctors do." Needless to say, this reply availed her nothing, and she quite properly was struck off the Roll. Probably she was well aware that expulsion was inevitable and decided to take this one chance of "getting her own back" by deliberate impertinence. At the same time it is possible that she may have met in her practice with doctors who do not take the temperature or do not look at the nurse's chart which shows it; for there are still to be found, especially in poor districts, medical men whose example in this respect is a disgrace to the profession. The number is, without doubt, steadily decreasing, but it is a great pity that there should still be any whose antiquated ideas and methods furnish ammunition of the kind quoted above to disgruntled midwives of the baser sort.

### THE SANDOW CASE.

The last echo of the series of commotions caused by the action taken by the General Medical Council against certain practitioners who were associated with the "Sandow Curative Institute" died away on June 5, when Mr. J. R. Wallace was struck off the Medical Register for continued contumacy.

With the merits of this particular case we have no wish to deal, but we cannot refrain from noting that since the matter was first taken up a great change has come over the business methods of the "Curative Institute." Formerly it was advertised so persistently and so universally that the bills must have amounted to thousands of pounds a year. Full pages in the daily press and folios in the monthly magazines confronted the public at every turn. This has lately all been abandoned, and we should like to think that the proceedings of the General Medical Council have been the lever in the change. Mr. Sandow's versatile energy is now being expended, we understand, upon the manufacture and sale of cocoa. Whether he took up this business because of diminished opportunities for healing the sick, or because he finds it the more profitable of the two, we do not inquire; but we congratulate him on having adopted a much more legitimate and unobjectionable outlet for his undoubted business capacity and enterprise.

### A WORD IN ADVANCE TO THE YORK CONGRESS.

There are some interesting points about the preliminary programme of the 27th Congress of the Royal Sanitary Institute, which is to be held at York from July 29 to August 3, under the patronage of Prince Arthur of Connaught, when the Archbishop of York will preside. The Congress will meet in five sections, which include sanitary science and preventive medicine; engineering and architecture; and domestic, infantile, and industrial hygiene. Among the sectional presidents are Sir Shirley Murphy, Mrs. Scharlieb, M.D., and Sir Thomas Oliver, M.D. During the Congress conferences will be held in which medical officers of health among others will take part. Professor Karl Pearson will lecture on "Eugenics and Public Health" and Professor H. R. Kenwood on "The Open Window." It will be noticed that every one of the subjects to be discussed has what may be termed a hospital side; that, in other words, each of the problems is from one point of view or another a hospital problem. We hope that this will not be forgotten at the Congress, and that, to take a typical example, those who treat the subject of architecture will not forget that hospital architecture is an exclusive and important branch of architecture generally, which by itself comprises a very large and expert field, and one, it is still necessary to add, which architects generally have been painfully slow to admit as a special subject. What the medical superintendent, for instance Dr. Mackintosh, M.V.O., has done for architecture, hospital superintendents can largely do for most, if not all, of the other subjects to be considered by the York Congress.

### A DOCTOR'S BEQUEST.

Dr. John Dixon Mann, M.D., F.R.C.P., of Harewood, Plymouth Grove, Manchester, whose death was recently recorded in our columns, and who was well known as Professor of Forensic Medicine and Toxicology at the University of Manchester, and examiner in these subjects to the Universities of Oxford, London, and Sheffield, has left estate valued at £24,639 gross, with net personalty £24,516. He bequeathed £1,000 each to the Salford Royal Hospital and to the University of Manchester, for the medical department, to be applied as the council may think fit. The residue of his property, which is expected to amount to £4,000 or £5,000, he left to the Salford Royal Hospital, to which, it will be remembered, he was physician.

### THE WERNHER MILLIONS.

The estate of the late Sir Julius Charles Wernher having been provisionally sworn at about £5,000,000, hospitals naturally observe with interest his list of bequests. King Edward's Hospital Fund receives £25,000, together with one-twelfth part of his residuary estate, such part upon trust to employ the income in making annual grants to such hospitals, present and future, as the governing body may think fit. The Kimberley Hospital, Kimberley, South Africa, is given £2,500, and the Bute Hospital, Luton, a similar sum. The London Hospital receives £5,000. The trustees, moreover, have discretionary power to pay out not more than £100,000 to such charities as they think fit, subject to certain conditions as to the mode and objects of distribution. On the whole, therefore, when they consider the large educational schemes—such as the foundation of a university at Groote Schuur, near Cape Town, for which £250,000 was bequeathed—which the testator had in view, the voluntary hospitals cannot complain of neglect in one of the largest estates of recent years.

### THE SUMMERS FAMILY AND ASHTON INFIRMARY.

The President of the Ashton District Infirmary, Alderman A. Shaw, J.P., has now received the £1,000 which Mrs. J. Summers, of Inglewood, Stalybridge, formerly promised to give in memory of her husband, the late Mr. John Summers. The name Summers, however, stands locally in no danger of being forgotten, particularly by the Ashton Infirmary, of which, indeed, the late Mr. Summers was once president. A Summers Ward records the family interest in the institution, and a brass tablet is to be affixed to it as a register of this gift. Mrs. Summers, moreover, has suggested that the sum alluded to should be devoted to the extension scheme, which thus helps the governors out of their difficulty by allowing a new laundry to replace the present building, which has earned its own supersession by some fifty years of work.

### A NEW FIELD FOR TESTATORS.

If hospital work is always bringing new sources of expenditure to light, and incidentally new risks with new treatments, it does not always find testators equally wide awake to the necessity of providing for them. Without entering the debatable ground of whether on the whole ear-marked bequests are to be encouraged or discountenanced, which must not be decided by the simple test of convenience alone, a recent bequest to the London Hospital deserves careful notice. Under the will of the late R. J. Winsor about £700 has been bequeathed to the London Hospital, the testator having read of the death of one of the hospital's radiographers. He left instructions that the money should be "applied towards the treatment and care of any patients who may through experimental work with $x$-rays or radium have suffered physical damage or hurt, or otherwise towards the treatment of any other patients in the hospital by means of $x$-rays or radium." In the first place it should be noticed that, though it was the death of a radiographer which brought the risk attaching to $x$-ray work to his notice, Mr. Winsor left his bequest for the benefit of injured patients, and not of injured radiographers. We venture to say that this proves careful and not careless thought on Mr. Winsor's part, and shall follow the further history of this bequest with interest.

### A NEW COTTAGE HOSPITAL IN SHROPSHIRE.

The foundation-stone of the new cottage hospital at Wellington, near Shrewsbury, which owes its foundation largely to the generous bequest of the late Mrs. Bowring, has now been laid by Lord Forester, whose family is well known in connection with the convalescent home at Llandudno and the hospitals at Broseley and Much Wenlock. Mr. H. Shepard, who is one of the trustees, recalled at the opening the terms of the bequest, by which a site of some three acres was left on which to build "The John Crump Bowring Hospital" for the urban area of Wellington, as well as £3,400 for its building and equipment. Mr. Shepard hopes that the wide discretion vested by the will in the trustees as to the class of patients which they may decide to admit may lead to the erection of shelters in the grounds for incipient phthisical patients. The Urban Council has been asked to appoint one of its number to the board of management, so that, as Mr. G. W. Harvey, chairman of the council, pointed out, the town will have a voice in its administration. Mr. Leslie T. Moore, A.R.I.B.A., is the architect, and it is to be noted that he is making a special study of this class of hospital construction.

### A SANATORIUM SECRETARY ON THE INSURANCE ACT.

A welcome, because original, criticism of the sanatoria provisions of the Insurance Act was made last week at the Manchester Conference of the National Association for the Prevention of Consumption by Dr. H. W. McConnell, who is honorary secretary of the Kelling Sanatorium at Holt. After suggesting that a patient should be taught that the arrest of his disease and a return to wage-earning capacity were the objects of his stay in a sanatorium, Dr. McConnell added that the Act ought to have provided for the creation of sanatorium and dispensary after-care committees. This, no doubt, is a gap, however difficult it may prove to fill, and it is one which is continually proving a source of anxiety to sanatorium superintendents. Among the exceedingly varied requests for help in the sphere of after-care which our Bureau of Information brings to light every week none is more characteristic and none was more promptly made than this: where can we find a suitable occupation for an arrested phthisical case? and, which is really complementary to it, what chance is there for an incipient case that cannot afford sanatorium fees to be employed as a paid servant? All experience teaches that a question which one person asks is of importance to a hundred who never ask it, and, even apart from the Insurance Act, which will magnify the problem, very little, if any, effort seems to have been made to co-ordinate after-care with sanatoria treatment. Dr. McConnell deserves public gratitude for insisting on the growing pressure of this problem.

### HOUNSLOW'S NEW HOSPITAL.

The foundation-stone of the new hospital for Hounslow and its district was laid on Saturday afternoon last by the Countess of Jersey. It is satisfactory to learn from the Chairman, the Rev. Henry Layton, that practically the whole of the £5,000 required for the erection of the hospital has already been subscribed. Designed by Mr. J. Ernest Franck, A.R.I.B.A., the hospital is intended to provide for the needs of the district for many years to come.

### LABOUR TROUBLES AND THE DRUG MARKET.

Business in the Drug market is still overshadowed by the labour troubles at the docks, which are still awaiting settlement. Transactions have been on a small scale, and of price-changes there have been few of any importance. Bromides are unchanged at the advanced prices recently noted, but a further rise in value should not take buyers unawares. The tendency in the Opium market has altered somewhat, the slightly downward movement in price which had been noticeable for some time having been checked for the moment as a result of reports from Smyrna to the effect that the new crop is not expected to turn out so well as had been anticipated. The general opinion, however, seems to be that the present value will not be maintained. There is practically no alteration in the position of morphine or codeine. There is no material change in the value of cod-liver oil, and it is not improbable that the low prices at present quoted will, with slight variations, be in force for some time; the demand is very quiet. Quicksilver has been slightly reduced in price, but as yet no alteration has been announced in the quotations for calomel, corrosive sublimate, and other salts of mercury. Chrysarobin is slightly cheaper. Buchu leaves still tend upwards in value. Until the transport strike is completely settled the Drug and Chemical markets cannot be expected to resume their normal appearance.

### TO OUR READERS.

Contributions are specially invited from any of our readers to these columns. They should deal with topical subjects and news. They must be authenticated for the information of the Editor only. The minimum payment if published is 5s. There is no hard-and-fast rule as to space, but notes of about twenty lines in length are preferred.

